# Predictors of unsuccessful pessary fitting in women with prolapse: a cross-sectional study in general practice

**DOI:** 10.1007/s00192-016-3107-4

**Published:** 2016-08-15

**Authors:** Chantal M. C. R. Panman, Marian Wiegersma, Boudewijn J. Kollen, Huibert Burger, Marjolein Y. Berger, Janny H. Dekker

**Affiliations:** Department of General Practice, University Medical Center Groningen, University of Groningen, P.O. Box 196, 9700 AD Groningen, The Netherlands

**Keywords:** General practice, Pelvic organ prolapse, Pessaries, Prediction, Risk factors

## Abstract

**Introduction and hypothesis:**

Pelvic organ prolapse is a common condition. There is inconsistency between predictors of unsuccessful pessary fitting in urological/gynaecological clinics. Research in general practice is scarce. The aim was to estimate the proportion of women in general practice with a symptomatic pelvic organ prolapse and unsuccessful pessary fitting, and to identify characteristics associated with unsuccessful pessary fitting.

**Methods:**

A cross-sectional study in general practice (*n* = 20) was carried out among women (≥55 years) with symptomatic prolapse (*n* = 78). Multivariate logistic regression analysis was used to identify predictors of unsuccessful pessary fitting.

**Results:**

In total, 33 women (42 %) had unsuccessful pessary fitting. Factors associated with unsuccessful pessary fitting were age (per year, OR 0.93 [95 % CI 0.87–1.00]), body mass index (per kg/m^2^, OR 1.14 [95 % CI 1.00–1.30]), and having underactive pelvic floor muscles (OR 2.60 [95 % CI 0.81–8.36]).

**Conclusions:**

Pessary fitting was successful in 58 %, indicating that pessary treatment may be suitable for many, but not for all women in general practice with symptomatic prolapse. The condition of the pelvic floor probably plays a role in the success of pessary fitting, as demonstrated by the association with underactive pelvic floor muscles, and body mass index. The association with age may reflect the higher acceptance of conservative treatments for prolapse in older women. This is the first study on predictive factors for unsuccessful pessary fitting in general practice. Therefore, further research should seek to confirm these associations before we can recommend the use of this information in patient counselling.

## Introduction

Pelvic organ prolapse is a common condition. In a community survey, 75 % of Dutch women aged 45–85 years had at least some degree of prolapse [1], and the prevalence of typical prolapse symptoms (i.e. seeing or feeling a vaginal bulge) has been reported to be about 3–12 % [2, 3]. Besides vaginal bulging, prolapse can cause a variety of pelvic floor symptoms, including a feeling of pelvic pressure or heaviness, pelvic pain, and urinary or faecal incontinence or obstruction [4]. In addition, prolapse can negatively affect daily activities, sexual function [5], and quality of life [6]. Prolapse is therefore a significant problem.

Treatment options for symptomatic prolapse are the insertion of a vaginal pessary, pelvic floor muscle training or reconstructive surgery. One year after treatment, research has shown no differences between pessary treatment and surgery in the improvement of urinary, bowel, or sexual function, in addition to quality of life parameters [7]. Reconstructive surgery is not always possible or desirable because of the high rates of comorbidity and frailty among older women, and the high risk of recurrence [8]. Pessary treatment can easily be offered in general practice, which is less expensive, generally closer to a patient’s home, and is generally easier to access compared with secondary care. Physicians can offer pessaries to the majority of patients with prolapse because there are very few contraindications. However, rates for successful pessary fitting range from 41 to 86 % [9–16], indicating that pessary treatment is not suitable for all women.

Research into the predictors of unsuccessful pessary fitting is scarce in general practice. Many studies have been performed in urological or gynaecological clinics, but their results cannot be extrapolated to general practice because of the potential for selection bias. In addition, the predictors of unsuccessful fitting have been inconsistent among studies. The predictors include a short vagina and wide vaginal hiatus [9, 13, 14], genital hiatus/total vaginal length (GH/TVL) ratio >0.8 [11], lower prolapse Pelvic Organ Prolapse Quantification system (POP-Q) stage [11], posterior wall prolapse [17], previous prolapse repair and hysterectomy [10, 12, 14–16], coexistent stress urinary incontinence [16], increased parity [10], age 65 years or younger [11], and smoking [11].

The scarcity of studies and the inconsistency of the results are surprising given that knowledge about the fitting rate and predictors of success are important to general practitioners when counselling patients about appropriate treatment. Further evidence is needed to facilitate an informed choice based on individualised estimates of the risk of failure. Therefore, we aimed to estimate the proportion of women in general practice with symptomatic prolapse at or beyond the hymen, who cannot be successfully fitted with a pessary. In addition, we aimed to identify the characteristics of those patients (including the findings during pelvic examination) that are associated with unsuccessful pessary fitting.

## Materials and methods

### Study design

We report on secondary analyses of data from a randomised controlled trial on the effects and cost-effectiveness of conservative treatments for pelvic organ prolapse in older women in general practice. The trial was conducted between October 2009 and December 2012 and was approved by the Medical Ethics Committee of the University Medical Centre Groningen, the Netherlands (METc2009.215). All participants provided written informed consent. An extensive description of the design is provided elsewhere [18].

### Participants

Women registered in a general practice in the northern part of the Netherlands (*n* = 20 practices), who screened positive for ≥1 pelvic floor symptoms related to prolapse on a postal questionnaire were invited for a clinical assessment. Pelvic floor symptoms included urinary incontinence, vaginal bulging, pelvic heaviness/pressure, or vaginal splinting required to start or complete micturition or defecation. During the clinical assessment women underwent a gynaecological examination using the POP-Q system to assess the degree of prolapse. In total, 162 women (aged ≥55 years) with a symptomatic prolapse, where the leading edge was at or beyond the hymenal remnants (advanced POP-Q stage 2 or stage 3), were randomised to pessary treatment or pelvic floor muscle training. This study only includes those women who were assigned to pessary treatment.

### Outcomes

The primary outcomes were the proportion of women with unsuccessful pessary fitting and the factors associated with unsuccessful pessary fitting.

### Pessary fitting procedure

A research physician who was trained in fitting pessaries chose the pessary size and format. The first choice was an open ring pessary, followed by a ring pessary with support. If a ring pessary could not be fitted, a Shaatz or Gellhorn pessary was tried. All pessaries were made of silicone (Milex, Chicago, IL, USA). A pessary was considered to be the correct size when the physician could place a single finger between the pessary and the vaginal wall, the prolapse was reduced to above the hymen, it felt comfortable to the patient, and it was retained during a Valsalva manoeuvre and coughing in both the supine and standing positions. After 2 weeks, an appointment was scheduled to evaluate the fit. Participants in whom the pessary fell out or who experienced discomfort within the first 2 weeks were refitted with a different type or size of pessary and reviewed again after another 2 weeks. A maximum of three attempts was made to achieve successful pessary fitting. Successful pessary fitting was defined as the ability to wear the pessary for 2 weeks without any discomfort, regardless of the number of pessary trials. Women were taught, if desired, to perform self-care of a pessary.

### Selection of predictors of unsuccessful pessary fitting

The selection of candidate predictors to be included in a prediction model was based on a review of the literature. The following potentially relevant predictors were considered for the multivariate logistic regression model: age (years), birth weight of the heaviest child given birth to (kg), hysterectomy (yes/no), other pelvic floor surgery (yes/no), body mass index (BMI; kg/m^2^), underactive or inactive pelvic floor muscles (yes/no) according to the International Continence Society (ICS) classification, POP-Q stage, genital hiatus (GH; cm), total vaginal length (TVL; cm) and the most prolapsed compartment (anterior wall, posterior wall or uterus/vault).

### Measurements

A standardized interview was conducted to collect data about patient characteristics, comorbidity, and the medical and obstetric history of the participants. Women underwent a urogynaecological examination using the POP-Q system [19] to assess the degree of prolapse and the GH and TVL. The function of the pelvic floor muscles was assessed by vaginal palpation in the supine position. Pelvic floor muscle function was examined by vaginal palpation of the pelvic floor muscles in the lithotomy position. Pelvic floor muscle function was categorised as normal, underactive, overactive, or inactive according to the classification system of the ICS. It was defined as normal when the voluntary contraction (VC) was normal/strong, the voluntary relaxation (VR) was complete, and both involuntary contraction (IC) and involuntary relaxation (IR) were present. Pelvic floor muscle function was defined as underactive when the VC was absent/weak, the VR was complete, the IC was absent/present and the IR was present. Overactive pelvic floor muscle function was defined as the VC being absent/weak/normal/strong, the VR being absent/partially present, and both the IC and IR being absent/present. Inactive pelvic floor muscle function was defined as an absent VC, a complete VR, and both the IC and IR were absent [20]. Four research physicians were trained by an experienced urogynecologist in performing the POP-Q measurement and to assess the pelvic floor muscle function. All clinical assessments were performed by one of these four research physicians. The Pelvic Floor Distress Inventory-20 (PFDI-20) [21] was used to assess pelvic floor symptoms, with higher scores indicating more distress. This questionnaire is divided into three subscales: the Pelvic Organ Prolapse Distress Inventory-6 (POPDI-6), for prolapse symptoms; the ColoRectal-Anal Distress Inventory-8 (CRADI-8), for colorectal/anal symptoms; and the Urinary Distress Inventory-6 (UDI-6), for urinary symptoms.

### Analyses

Multivariate logistic regression analysis was used to identify independent predictors of unsuccessful pessary fitting. A manual backward elimination approach with all candidate predictors followed to arrive at a model that included only the strongest predictors. We used the Akaike Information Criterion, which corresponds to a *p* value of ≥ 0.157 as the criterion for removal from the model [22]. Calibration of the multivariate model, or the extent of agreement between the predicted and observed unsuccessful fitting, was evaluated using the Hosmer–Lemeshow goodness-of-fit test. A calibration plot was created to display the concordance between the observed and predicted probabilities of unsuccessful pessary fitting. Discriminatory performance, or the ability of the model to distinguish between women with successful and unsuccessful pessary fitting was evaluated by the area under the receiver operating characteristics (ROC) curve. Odds ratios (ORs) and 95 % confidence intervals (CIs) are given, unless otherwise stated. All statistical analyses were performed using IBM SPSS for Windows, version 22.0 (IBM, Armonk, NY, USA).

## Results

### Patients

Of the identified participants, 3 women were excluded because there were missing values for birth weight of the heaviest child (*n* = 2) and the most prolapsed compartment (*n* = 1), leaving 78 women for the analysis (<5 % incomplete cases). The characteristics of the study population are presented in Table 1.

**Table 1:** Characteristics of the study population: women with successful and unsuccessful pessary fitting (*n* = 78)

Characteristics	Successful pessary fitting *n* = 45 (58.0 %)	Unsuccessful pessary fitting *n* = 33 (42.0 %)
Age, years, median (IQR)	65.9 (59.5–71.3)	63.3 (57.9–67.0)
Body mass index, kg/m^2^, median (IQR)	25.3 (22.5–28.1)	26.7 (24.3–28.1)
Parity, median (IQR)	2.0 (2.0–3.0)	2.0 (2.0–3.0)
Education level, *n* (%)		
Lower education	21 (47)	12 (36)
Intermediate education	8 (18)	11 (33)
Higher education	16 (36)	10 (30)
Charlson Index, median (IQR)	0 (0–1)	0 (0–0.5)
Sexually active, *n* (%)	18 (40)	23 (70)
Having a partner, *n* (%)	34 (76)	27 (82)
Smoking, *n* (%)	21 (47)	18 (55)
Birthweight of heaviest child, median (IQR)	3.8 (3.2–4.0)	4.0 (3.5–4.5)
Hysterectomy, *n* (%)	7 (16)	9 (27)
Other pelvic floor surgery, *n* (%)	4 (9)	4 (12)
≥1 first-degree relative with prolapse, *n* (%)	16 (36)	21 (64)
PFDI-20 score, median (IQR)	54.2 (28.1–77.1)^a^	61.5 (45.3–96.1)^b^
POPDI-6 score, median (IQR)	12.5 (8.3–25.0)	16.7 (8.3–33.3)^b^
CRADI-8 score, median (IQR)	12.5 (0.8–18.8)^b^	18.8 (7.1–29.7)
UDI-6 score, median (IQR)	20.8 (12.5–36.5)^b^	29.2 (18.8–41.2)
Most prolapsed compartment, *n* (%)		
Anterior wall	36 (80)	29 (88)
Posterior wall	7 (16)	4 (12)
Uterus/vault	2 (4)	0 (0)
Underactive/inactive pelvic floor muscles, *n* (%)	31 (69)	26 (79)
GH, cm, median (IQR)	3.0 (2.0–3.0)	3.0 (2.0–4.0)
TVL, cm, median (IQR)	9.0 (8.0–10.0)	9.0 (8.0–10.0)^b^

*PFDI-20* Pelvic Floor Distress Inventory-20 (range 0–300), *POPDI-6* Pelvic Organ Prolapse Distress Inventory-6 (range 0–100), *CRADI-8* ColoRectal-Anal Distress Inventory-8 (range 0–100), *UDI-6* Urinary Distress Inventory-6 (range 0–100), *GH* genital hiatus, *TVL* total vaginal length

^a^Two missing items

^b^One missing item

In total, 45 women (58 %) had a successful fitting. Of these, 30 women were fitted with an open ring pessary, 14 with a ring pessary with support, and 1 with a Shaatz pessary. In the remaining 33 women (42 %), pessary fitting was unsuccessful because it was not possible to find a properly fitting pessary in 21 women (64 %). Patient-related reasons were the cause of non-fitting in 12 women (36 %): an increase in or the development of urinary incontinence (*n* = 4), emotional resistance to pessary fitting/treatment (*n* = 4), discomfort during intercourse (*n* = 2), increased vaginal discharge (*n* = 1; the patient found it bothersome and was unwilling to use topical oestrogens), and urinary tract infection (*n* = 1; the patient related the infection to the pessary treatment). Median (interquartile range) number of consultations was 1.0 (1.0–2.0) for women with successful pessary fitting and 2.0 (2.0–3.0) for women with unsuccessful fitting.

### Multivariate analysis of unsuccessful pessary fitting

From the selected candidate predictors, GH and TVL were excluded since all patients in the study had a TVL >6 cm and a GH < 5 cm. The candidate predictors “hysterectomy” and “other pelvic floor surgery” were combined into one variable (pelvic floor surgery) to enlarge the number of cases for this variable. Table 2 shows the results of the final multivariable logistic regression analysis, including all variables with a p-value <0.157. Variables in this final model were age (per year; OR 0.93 [95 % CI 0.87–1.00]), BMI (per kg/m^2^; OR 1.14 [95 % CI 1.00–1.30]), and underactive or inactive pelvic floor muscles (OR 2.60 [95 % CI 0.81–8.36]). Calibration of the final multivariable model was evaluated with the Hosmer and Lemeshow goodness-of-fit test (*p* = 0.81); the calibration plot displayed concordance between the observed and predicted probabilities of unsuccessful pessary fitting (plot not shown). Discrimination of the final model was evaluated with the area under the ROC curve (0.69 [95 % CI 0.57–0.80]).

**Table 2:** Predictors of unsuccessful pessary fitting after multivariate logistic regression (*n* = 78)

Variables	Univariate LR^a^, OR (95 % CI)	Final model after MLR, OR (95 % CI)	MLR, *p* value
Age (per year)	0.94 (0.88–1.00)	0.93 (0.87–1.00)	0.046
Birthweight of heaviest child (per kg)	1.80 (0.86–3.77)		
Pelvic floor surgery (versus no pelvic floor surgery)	1.31 (0.46–3.7)		
Body mass index (per kg/m^2^)	1.09 (0.97–1.23)	1.14 (1.0–1.30)	0.056
Underactive/inactive pelvic floor muscles (versus normal pelvic floor muscle function)	1.68 (0.59–4.78)	2.60 (0.81–8.36)	0.11
POP-Q stage 3 (versus stage 2)	0.96 (0.36–2.55)		
Anterior wall prolapse (versus posterior wall)	1.41 (0.38–5.29)		

*LR* logistic regression analysis, *MLR* multivariable logistic regression analysis

^a^Univariate associations of all candidate predictors in the full model. A manual backward elimination approach with all these candidate predictors in the model was followed to arrive at a final model that included only the strongest predictors. The Akaike Information Criterion (*p* value of ≥ 0.157) was used as the criterion for removal from the model

## Discussion

### Main findings

In this study in women in general practice with symptomatic pelvic organ prolapse at or beyond the hymen, pessary fitting (typically open ring) was unsuccessful in 42 % (33 out of 78). The success rate of 58 % is consistent with previous reported successful fitting rates of 41–86 % [9–16].The factors associated with unsuccessful pessary fitting in this study were lower age, higher BMI, and underactive or inactive pelvic floor muscles.

### Strengths and limitations

To our knowledge, this is the first study about predictors of unsuccessful pessary fitting in general practice. The use of multivariate logistic regression to identify independent predictors for unsuccessful fitting elaborates on existing studies based on univariate regression analysis, which do not provide information on independent relationships [9, 13–15].

When interpreting the results of this study, potential study limitations should be taken into account. Characteristics that were associated with unsuccessful pessary fitting, vary between studies, with few characteristics identified consistently. This complicated the pre-selection of the most important candidate predictors for our prediction model. The rule of thumb that logistic regression models should be used with a minimum of 10 events per predictor variable (EPV) is based on very few simulation studies in which only the numbers of events was varied [23]. Vittinghoff and McCulloch [23] conducted a large simulation study of other influences on confidence interval coverage, type I error, relative bias, and other model performance measures. They concluded that the rule of thumb of a minimum of 10 events per variable can be relaxed based on the finding that model performance problems were uncommon with 5–9 EPV, and still observed with 10–16 EPV. Based on this, and the exploratory nature of our study, we decided to use 5 EPV. In addition, the Akaike Information Criterion (*p* ≥ 0.157) was used as the criterion for removal from the model to prevent the possibility that a true predictor would be missed and to reduce the extent of overfitting (i.e. over-optimism of the model) [22, 24].

### Interpretation

In our study of women aged 55 years and older, higher age was negatively associated with unsuccessful pessary fitting in the final multivariate logistic regression model, which means that in our study, which was explorative, the risk of unsuccessful pessary fitting decreased with advancing age. Evidence of the association is conflicting: some studies did not find an association between age and the likelihood of unsuccessful pessary fitting (e.g. Clemons et al., Markle et al., Mutone et al., Nguyen and Jones [9, 14–16]), whereas others found age 65 years or younger to be a predictor of unsuccessful pessary fitting [11]. Older women are more likely to choose pessary treatment over reconstructive surgery [25, 26] and older age has been shown to be a good predictor of the continuation of pessary use in successfully fitted patients [27, 28]. This may indicate that the acceptance and appreciation of pessary treatment for prolapse is higher in older women. Health care providers could therefore try pessary treatment in women of all ages, but especially in older women.

In our study, a higher BMI was associated with unsuccessful fitting. This has been found before [15]. An explanation for this might be that increased pressure on the pelvic area in women with a high BMI impedes pessary fitting. However, some studies report a lack of association of BMI with unsuccessful pessary fitting [12, 14]. Thus, this association needs further exploration.

Women with underactive or inactive pelvic floor muscles, compared with those with normal pelvic floor function, had a higher likelihood of unsuccessful fitting. It is conceivable that underactive pelvic floor muscles provide insufficient support to the pessary, which in turn results in the pessary falling out. This finding is different from the results of the two other studies that looked at the role of pelvic floor muscle strength in pessary fitting and in which no association was found [11, 12]. A possible explanation for these conflicting results might be the differences in the definition and measurement of underactive pelvic floor muscles.

We did not find an association between the severity of pelvic organ prolapse and the likelihood of an unsuccessful pessary fit. We did not find an association between the most prolapsed compartment (anterior wall, posterior wall, or uterus/vault) and unsuccessful pessary fitting either. Although this is in accordance with previous studies [9, 14, 15], our study may have lacked the power to detect an association because of the low number of patients with the posterior wall as the leading edge of the prolapse (*n* = 11). So far, there is controversy regarding the effectiveness of a pessary for posterior wall prolapse as a pessary uses the pelvic floor as a support base and its supportive effect was therefore thought to be presumably anteriorly. To date, there is insufficient evidence to state that a posterior wall prolapse is a risk factor for unsuccessful pessary fitting. The effect of a wide GH or a short vagina on pessary fitting could not be studied because of a lack of patients with these conditions in our sample. In primary care, women with a prolapse often have a normal hiatus width and vaginal length. Patients referred for secondary or tertiary care settings may more frequently show a hiatus > 5 cm or a vaginal length < 6 cm.

The discriminatory performance of our final multivariate regression model was moderate and the calibration seemed to be good. Information about the discrimination and calibration is relevant to estimate the performance of a prediction model. This study was performed to generate hypotheses about predictive factors for unsuccessful pessary fitting in general practice rather than to arrive at a prediction model to be used in clinical practice. Information about the independent risk factors for unsuccessful pessary fitting is therefore of greater interest than the performance of the final multivariate logistic regression model. Future research should address the observed risk factors and seek to confirm these associations, preferably in a larger population. Until then, the association between the identified predictors and fitting failure is insufficiently strong to advise against pessary use in women with those predictors.

## Conclusions

This study showed that pessary fitting was successful in 58 % of women in general practice with a symptomatic prolapse. This indicates that pessary treatment may be suitable for many, but not all women with a symptomatic prolapse. This study was performed to generate hypotheses about independent risk factors for unsuccessful pessary fitting in general practice. Our results indicated that lower age, higher BMI and underactive or inactive pelvic floor muscle function were associated with a higher risk of unsuccessful pessary fitting. As this was to our knowledge the first study on predictive factors for unsuccessful pessary fitting in general practice, further research is needed to confirm the associations we found and prediction models have to be validated in other primary care populations. Only then can we use predictors of fitting failure when counselling women about pessary use.
